# Validation of PHASE for deriving N-acetyltransferase 2 haplotypes in the Western Cape mixed ancestry population

**DOI:** 10.4102/ajlm.v9i1.988

**Published:** 2020-12-17

**Authors:** Celeste Swart, Surita Meldau, Chad M. Centner, Adrian D. Marais, Fierdoz Omar

**Affiliations:** 1 Faculty of Health Sciences, University of Cape Town, Cape Town, South Africa; 2 National Health Laboratory Service (NHLS), Groote Schuur Hospital, Cape Town, South Africa; 3 Division of Chemical Pathology, University of Cape Town, Cape Town, South Africa; 4 Division of Medical Microbiology, University of Cape Town, Cape Town, South Africa; 5 National Health Laboratory Service (NHLS), Medical Microbiology, Groote Schuur Hospital, Cape Town, South Africa; 6 Division of Chemical Pathology, University of Cape Town, Cape Town, South Africa

**Keywords:** *NAT2*, haplotyping, isoniazid, pathology, molecular

## Abstract

**Background:**

There is a shortage of data on the accuracy of statistical methods for the prediction of N-acetyltransferase 2 (*NAT2*) haplotypes in the mixed ancestry population of the Western Cape.

**Objective:**

This study aimed to identify the *NAT2* haplotypes and assess the accuracy of PHASE version 2.1.1 in assigning *NAT2* haplotypes to a mixed ancestry population from the Western Cape.

**Methods:**

This study was conducted between 2013 and 2016. The *NAT2* gene was amplified and sequenced from the DNA of 100 self-identified mixed ancestry participants. Haplotyping was performed by molecular and computational techniques. Agreement was assessed between the two techniques.

**Results:**

Haplotypes were assigned to 93 samples, of which 67 (72%) were ambiguous. Haplotype prediction by PHASE demonstrated 94.6% agreement (kappa 0.94, *p* < 0.001) with those assigned using molecular techniques. Five haplotype combinations (from 10 chromosomes) were incorrectly predicted, four of which were flagged as uncertain by the PHASE software. Only one resulted in the assignment of an incorrect acetylation phenotype (intermediate to slow), although the software flagged this for further analysis. The most common haplotypes were NAT2*4 (28%) followed by NAT2*5B (27.4%), NAT2*6A (21.5%) and NAT2*12A (7.5%). Four rare single nucleotide variants (c.589C>T, c.622T>C, c.809T>C and c.387C>T) were detected.

**Conclusion:**

PHASE accurately predicted the phenotype in 92 of 93 samples (99%) from genotypic data in our mixed ancestry sample population, and is therefore a suitable alternative to molecular methods to individualise isoniazid therapy in this high burden tuberculosis setting.

## Introduction

Tuberculosis remains a global life-threatening infectious disease.^1^ Patients in developing countries are particularly vulnerable, as informal settlements and overcrowded environments provide optimal conditions for the spread of tuberculosis. According to the National Strategic Plan, South Africa has the third highest tuberculosis burden worldwide. with an annual incidence of approximately 1%.^2^ The Western Cape demonstrates nearly the highest global incidence of tuberculosis, with the mixed ancestry population being particularly affected.^3^

Isoniazid is a key first-line antimicrobial drug in the treatment of tuberculosis. The dosage of isoniazid is usually standard and, in most settings, related to body mass in accordance with World Health Organization treatment guidelines.^4^ However, elimination of, and thus response to, the drug are affected by the patient-specific expression of metabolising enzymes, in particular arylamine N-acetyltransferase 2 (*NAT2*), which catalyses N-acetylation of aryl amines and aryl hydrazines.^5^ These drugs include clinically important antimicrobial agents such as isoniazid and sulphonamides, caffeine, dapsone as well as some toxigenic substances and carcinogenic chemicals derived from the environment and human diet. Expression of *NAT2* is limited to the intestine and liver.^5^

The *NAT2* gene is located on chromosome 8p22 and has two exons,^6,7,8^ one of which is transcribed.^9^ Since the discovery of *NAT2* in 1990,^10^ extensive studies in various population groups have linked *NAT2* genetic variants to enzyme function, classifying individuals as either slow, intermediate or fast acetylators. The different acetylator phenotypes determine individual responses to toxins and prescribed medications, and also influence cancer susceptibility.^11^ The acetylation phenotype can affect clinical outcome on standard drug doses and may result in an increased risk of therapeutic failure or adverse drug reactions.^12^

Both lack and excess of *NAT2* (slow and fast acetylation) can be associated with isoniazid hepatotoxicity. The major metabolites associated with isoniazid metabolism are thought to be responsible for isoniazid-induced liver injury, while isoniazid itself can also cause immune-mediated hepatotoxicity by binding to liver proteins and serving as a hapten.^13^ It is thus postulated that fast acetylators may have a poor response to treatment as a result of suboptimal exposure to isoniazid due to reduced isoniazid plasma levels, as well as liver injury due to an increased rate of isoniazid metabolite formation.^13^ Conversely, slow acetylators achieve higher isoniazid plasma levels, since they cannot efficiently metabolise the drug, leading to hepatic injury.^13^

Distinct *NAT2* haplotypes have been described, consisting of up to four single nucleotide variants (SNVs) in various combinations. Over 100 haplotypes have been classified into 20 groups according to defining mutations, for example, the NAT2*5 group includes 29 different haplotypes and is defined by the c.341T>C SNVs. Each haplotype is associated with an acetylation phenotype. In human populations globally, the seven most prevalent *NAT2* haplotypes are: NAT2*4, NAT2*5B, NAT2*6A, NAT2*7B, NAT2*12A, NAT2*13A and NAT2*14.^14^ These haplotypes include various combinations of *NAT2* SNVs.^15^ NAT2*4 is classified as the wild type and is associated with fast (normal) acetylator status, as are the NAT2*12A (c.803A>G) and NAT2*13A (c.282C>T) haplotypes. The other four common haplotypes are associated with either significantly decreased expression or decreased stability of the *NAT2* enzyme.^16^ Heterozygotes for fast and slow alleles demonstrate intermediate acetylating activity.

Genotyping is typically performed using conventional restriction fragment length polymorphism (RFLP) analysis to detect the most common variants only, or Sanger sequencing to detect all SNVs in the *NAT2* region. Ambiguous *NAT2* genotyping results (the presence of more than one heterozygous SNV in the same patient) commonly arise from sequencing data due to simultaneous analysis of both maternal and paternal alleles. This can cause uncertainty predicting the *NAT2* phenotype as it may be unclear if one or both alleles carry variants with functional effects. In these cases, more complex molecular techniques are required in addition to Sanger sequencing for complete and accurate haplotyping.^17^ These include RFLP or amplification-refractory mutation system (ARMS) amplification.^18^ These methods are tedious for implementation in a routine diagnostic laboratory, and therefore computational haplotype reconstruction programs have been developed that statistically predict the most likely haplotype present using algorithms that take into account established allele frequencies within the population. The computational algorithms offer a quicker, cheaper and more practical alternative. Several approaches are available, including Clark’s algorithm, the Expectation Maximum algorithm and the Bayesian implementation, which estimate the maximum likelihood of haplotype frequencies.^19^ The software program PHASE (used in this study) is considered to be the reference standard for computational haplotype inference.^20,21^ It utilises the Bayesian approach for reconstructing haplotypes based on population genotype data. Using prior information (based on beliefs about patterns of haplotypes expected in certain population samples), as well as the information obtained from the observed data, the most likely haplotype is estimated and reconstructed for each allele in each individual.^22^

The proportions of slow and fast acetylators differ among various populations. In addition, the effect of different acetylation phenotypes on isoniazid-induced hepatic toxicity has been shown to be inconsistent across ethnic groups.^23^ There is limited *NAT2* genotypic data in African populations; the few known demonstrate nucleotide variants that differ from those commonly described in European populations. African populations are genetically diverse,^24^ and data can therefore not be extrapolated among the various population groups.

The South African mixed ancestry population (8.9% of the South African population) is described as a distinct ethnic group with a complex genetic admixture,^25^ with over 60% living in the Western Cape and particularly in the Cape Town area. The major ancestral populations contributing to the genetic diversity of this group include Khoisan and non-Khoisan Africans, with smaller contributions from both European and Indian Asian descent populations.^26^ The genotypic complexity and origin of this population not only influence disease susceptibility,^27^ but may also have implications when computational haplotype prediction algorithms are employed.

It would be ideal to determine the *NAT2* acetylation status of each patient in order to individualise isoniazid therapy. However, the complex manual molecular haplotyping techniques are expensive, time-consuming and tedious,^17^ and are thus not realistically feasible in a high tuberculosis burden setting such as the Western Cape where this study was performed. While the PHASE computational algorithm is used routinely at many centres, its performance has not been validated in the South African mixed ancestry population.

This study aims to describe the *NAT2* haplotypes identified in this population and to assess the accuracy of PHASE version 2.1.1 in assigning *NAT2* haplotypes in the mixed ancestry population of the Western Cape.

## Methods

### Ethical Considerations

The Human Research Ethics Committee (HREC REF: 327/2014), appointed by the Faculty of Health Sciences at the University of Cape Town, approved this study. All the work conducted was performed on pre-extracted, stored DNA from participants who gave informed consent for research conducted in a previous study, as well as for any future molecular testing. The DNA samples were selected at random from participants of self-identified mixed ancestry by allocation of numbers only.

### Study site and population

This study was conducted between 2013 and 2016. The blood samples (*n* = 100) used in this study were sourced with informed consent from randomly selected, self-identified mixed ancestry adult patients attending the Lipid outpatients’ clinic at Groote Schuur Hospital in Cape Town, South Africa. DNA was extracted and stored.

### Molecular techniques

The *NAT2* gene was amplified by polymerase chain reaction (PCR), sequenced (using Sanger sequencing) and haplotyped using molecular and computational techniques as described below.

#### Polymerase chain reaction

The PCR was performed using primers designed to flank the entire coding region of the *NAT2* gene (PCR amplicon size 1003 bp). The reaction consisted of the appropriate forward (5′GTCACACGAGGAAATCAAATGC3′) and reverse (5′AGTTGATAATTAGTGAGTTGGGTGA3′) *NAT2* primers (0.5 *µ*M), deoxynucleotide triphosphates (0.2 mM), Supertherm Gold taq DNA polymerase (0.75 U) (JMR Holdings, Kent, United Kingdom), 1 X buffer containing MgCl_2_ at 1.5 mM final concentration (supplied) and approximately 50 ng to 150 ng DNA. Polymerase chain reaction cycling conditions were as follows: 94 °C for 3 minutes; 35 cycles of: 94 °C for 30 s, 56 °C for 30 s and 72 °C for 40 s, followed by 72 °C for 8 min. Reaction efficacy was assessed by agarose gel electrophoresis (using 2% agarose gel with the addition of ethidium bromide for visualisation) prior to further analysis.

#### Sequencing of polymerase chain reaction products

**FIGURE 1:**
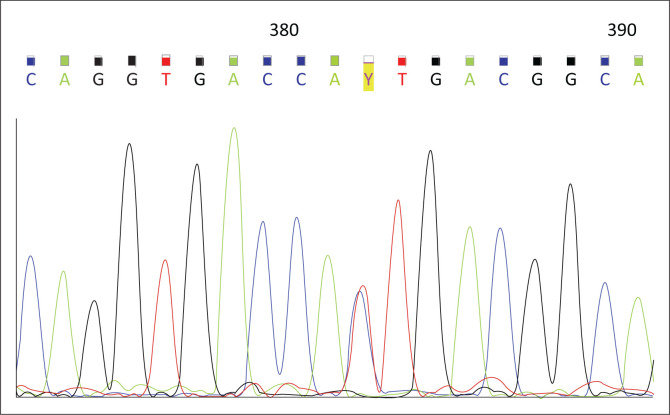
Electropherogram demonstrating heterozygosity for the c.341T>C (p.I114T) single nucleotide variation, Western Cape, South Africa, 2013–2016. Single nucleotide variants depicted by the red ‘Y’ highlighted in yellow.

**FIGURE 2:**
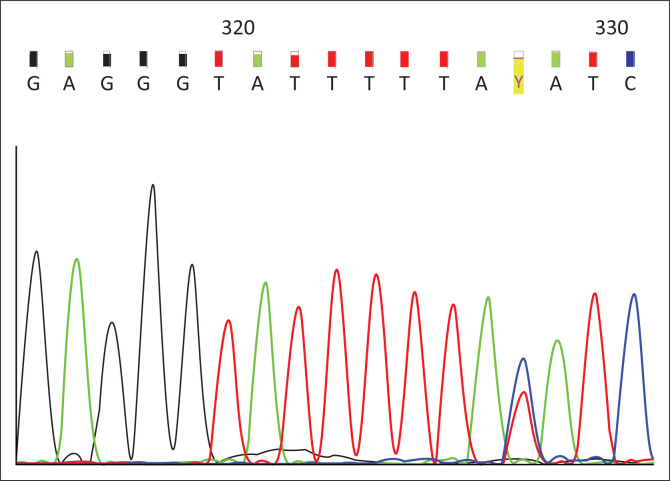
Electropherogram demonstrating heterozygosity for the c.282C>T (p.Y94Y) single nucleotide variation, Western Cape, South Africa, 2013–2016. Single nucleotide variants depicted by the red ‘Y’ highlighted in yellow.

Sequencing of the resulting PCR products were outsourced to the Central Analytical Facility (Stellenbosch University, South Africa). Forward and reverse sequencing were performed. The sequencing data were compared to the *NAT2* genomic reference sequence obtained from GenBank (NG_012246.1) (https://www.ncbi.nlm.nih.gov/genbank/) to identify all *NAT2* gene variants present (Figure 1 and Figure 2).

#### Restriction fragment length polymorphism

**FIGURE 3:**
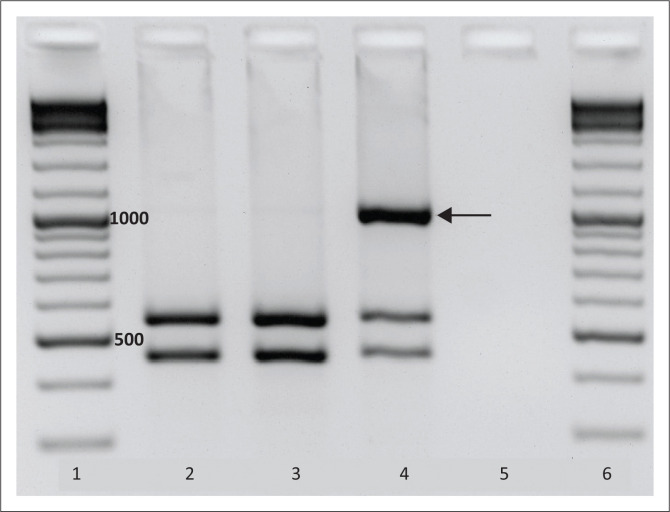
*Kpn1* restriction fraction length polymorphism for c.481C>T wildtype and heterozygous variant, Western Cape, South Africa, 2013–2016. Agarose gel electrophoresis of *NAT2* PCR products digested with *Kpn1* restriction enzyme. The c.481C>T variant destroys a *Kpn1* recognition site. Lanes 1 and 6: DNA ladder (Thermo Scientific GeneRuler DNA Ladder Mix) with relevant corresponding bp sizes indicated. Lane 2: Patient sample (no c.481C>T variation). Lane 3: Normal control (no c.481C>T variation). Lane 4: Heterozygous control (c.481C>T variation on one allele). Lane 5: Blank. The arrow demonstrates the uncut allele (1003 bp) containing the c.481C>T single nucleotide variant. The two bands (553 base pairs and 450 base pairs) below this band demonstrate the normal (and therefore cut) allele. The restriction site on the normal allele is recognised and therefore cut into smaller fragments, while the affected allele is not digested, lending itself to excision for single allele sequencing.

Restriction fragment length polymorphism was used to separate individual alleles in 25 samples with ambiguous haplotypes. The c.481C>T and c.191G>A variations destroy cleavage sites for *Kpn*1 and *Msp*1. *Kpn*1 was therefore used in samples heterozygous for c.481C>T (*n* = 22), while *Msp*1 was used in those with heterozygous c.191G>A (*n* = 3). While the normal allele is recognised and digested into two smaller fragments, the affected allele is not (Figure 3). Reaction products were separated by agarose gel electrophoresis (2%) followed by excision, agarose gel extraction and sequencing of the undigested allele.

#### Amplification-refractory mutation system polymerase chain reaction

Amplification-refractory mutation system PCR was performed for the remaining cases with ambiguous haplotypes containing heterozygous c.282C>T SNVs (*n* = 42). An ARMS forward primer (5′CCACAATGTTAGGAGGGTATTTTTAT3′) was designed to recognise and amplify only alleles containing the c.282T variant base. Polymerase chain reactions were performed as before, using the same reverse primer, with the exception of an annealing temperature of 62 °C. After checking the reaction efficacy with agarose gel electrophoresis, the individual alleles were sequenced as before.

### Data analysis

Sequence data were aligned to published reference sequences using the ClustalW alignment function in BioEdit version 7.2.5^28^ and analysed. The Court Lab online calculator was used to assess whether the variants were in Hardy-Weinberg equilibrium,^29^ and haplotypes were assigned by molecular methods.

### PHASE computational algorithm

Results obtained from genotyping of both ambiguous (*n* = 67) and non-ambiguous samples (*n* = 26) were used as input data for the PHASE computational algorithm.^21,22^ The following data were inputted: number of variant positions, variant nucleotide positions and the genotypes for each sample at those positions (wild type, heterozygous, homozygous). The following parameters were specified: number of iterations (= 1000), thinning interval (= 1), burn-in (= 100).

### Statistical analysis

The information obtained by molecular and computational haplotyping was compared by kappa statistics using Stata 13 (StataCorp LLC, College Station, Texas, United States). The *p*-values less than 0.05 were considered statistically significant.

## Results

Genotypes from all 100 samples were found to be in Hardy-Weinberg equilibrium for each SNV detected in the study population (Table 1). Four samples were heterozygous for both c.590G>A and c.857G>A, while another one was heterozygous for both c.341T>C and c.803A>G. No further work was done on these, although future studies should aim to analyse these with similar ARMS or RFLP techniques and those employed here. An additional two ambiguous samples could not be assigned haplotypes due to degraded DNA and repeatedly failed amplification – a total of seven samples were thus excluded from further analysis.

**TABLE 1:** Frequencies of heterozygotes and homozygotes for each observed single nucleotide variant, with associated Hardy-Weinberg equilibrium probabilities, Western Cape, South Africa, 2013–2016.

Variant position	Heterozygous (*n*)	Homozygous (*n*)	*p*
191 G>A	3	0	0.878
282 C>T	45	10	0.790
341 T>C	44	10	0.910
387 C>T	2	0	0.910
403 C>G	2	0	0.910
481 C>T	42	8	0.840
589 C>T	1	0	0.960
590 G>A	40	3	0.190
622 T>C	1	0	0.960
803 A>G	52	13	0.350
809 T>C	1	0	0.960
857 G>A	12	0	0.520

Note: Sample size = 100.

Of the 93 remaining samples, 26 were non-ambiguous genotypes and therefore did not require haplotyping. These 26 genotypes comprised either only wild type variants (*n* = 6), or were homozygous for any particular haplotype (*n* = 20). Separation of the non-ambiguous alleles by RFLP or ARMS PCR was thus not necessary and haplotypes could be assigned directly to these samples. Allele separation and haplotype determination for the remaining 67 samples were obtained using the RFLP and ARMS methods described earlier. The non-ambiguous samples demonstrated a predominance of haplotype NAT2*5B (28%), followed by NAT2*4 (25%), NAT2*6A (17%), NAT2*12A (12%), NAT2*13A and NAT2*5C (both 6%), NAT2*5A (4%) and NAT2*27 (2%).

Apart from the commonly described *NAT2* SNVs, four additional variants were detected in our study population. A single sample was heterozygous for the c.622T>C (p.Tyr208His) variant (rs56387565). In this instance, it co-occurred with c.803A>G, resulting in the less common haplotype NAT2*12F. Another was heterozygous for c.809T>C (p.Ile270Thr) (rs868725509). It co-occurred with c.341T>C and c.803A>G; molecular haplotyping could not be performed on this ambiguous sample due to the inability to delineate with available methodology. A single nonsense variant, c.589C>T (p.Arg197*), resulting in a stop codon, was detected in isolation in one patient.^30^ Two samples were heterozygous for a very rare variant, c.387C>T (p.Ser129=) (rs144828000). In both of our patients, it co-occurred with the c.282C>T and c.590G>A variants (NAT2*6A).

The most common haplotypes in the 67 ambiguous samples, derived by molecular methods, were NAT2*4 (29.1%) followed by NAT2*5B (26.9%), NAT2*6A (23.1%) and NAT2*12A (6%). These frequencies were similarly predicted by PHASE (Table 2). Of 67 ambiguous samples, 15 (22%) required further molecular testing (i.e. RFLP or ARMS PCR) to differentiate between slow and intermediate acetylation phenotypes. In 14 (93%) cases, the ambiguous genotype was resolved to predict a slow acetylation phenotype.

**TABLE 2:** Haplotype frequencies as derived by molecular methods, as well as predicted by the PHASE computational algorithm, Western Cape, South Africa, 2013–2016.

NAT2*	Total haplotypes†		Ambiguous haplotypes			
	2n‡ Molecular (93 samples)		2n‡ Molecular (67 samples)		2n‡ PHASE (67 samples)	
	
	*n*	%	*n*	%	*n*	%
NAT2* 4	52	28.0	39	29.1	41	30.6
NAT2* 5A	5	2.7	3	2.2	2	1.5
NAT2* 5B	51	27.4	36	26.9	38	28.4
NAT2* 5C	5	2.7	2	1.5	2	1.5
NAT2* 6A	40	21.5	31	23.1	32	23.9
NAT2* 6P	1	0.5	1	0.7	0	0.0
NAT2* 7B	7	3.8	7	5.2	7	5.2
NAT2* 12A	14	7.5	8	6.0	5	3.7
NAT2* 12F	1	0.5	1	0.7	1	0.7
NAT2* 13A	5	2.7	2	1.5	1	0.7
NAT2* 14A	0	0.0	0	0.0	1	0.7
NAT2* 14B	2	1.1	2	1.5	0	0.0
NAT2* 14C	1	0.5	1	0.7	0	0.0
NAT2* 14G	0	0.0	0	0.0	2	1.5
NAT2* 24	1	0.5	1	0.7	2	1.5
NAT2* 27	1	0.5	0	0.0	0	0.0

* , Standard nomenclature in Human *NAT2* haplotypes.

† , Seven haplotypes not available.

‡ , Two chromosomes per participant.

There was a 94.6% agreement between the haplotypes obtained by molecular methods and those predicted by PHASE, with a strong correlation (kappa 0.94, *p* < 0.001). Five haplotype combinations (from 10 chromosomes) were incorrectly predicted. Of these, only one resulted in a change of acetylation phenotype (intermediate to slow). Furthermore, the program flagged four of these five incorrect haplotype combinations (including the one incorrectly assigned to slow acetylator status) to indicate that uncertainty was present around one of the predicted variant positions.

## Discussion

*NAT2* genotypes were obtained by molecular methods for 100 samples. Ninety-three genotypes were then used to validate the accuracy of PHASE in predicting haplotypes. Twenty-six of these samples had non-ambiguous genotypes; of the remaining 67 samples, only 15 (22.5%) technically needed further allelic haplotyping, where the ambiguous genotype could lead to ambiguous acetylation phenotype prediction. This is in keeping with possible clinical scenarios, where non-ambiguous samples would not in fact need to be subjected to PHASE analysis to obtain haplotypes.

Four rare SNVs were detected in our study population. The variant at position c.622T>C (rs56387565) results in a non-synonymous change (p.Tyr208His); the functional effect of this variant is not well studied, although it has been linked to slow acetylation according to dbSNP.^31^ Another variant at c.809T>C (rs868725509) is also non-synonymous (p.Ile270Thr) and has not been characterised. The nonsense variant c.589C>T (p.Arg197*) results in a stop codon with a subsequent presumed lack of functional protein expression.^30^ These three SNVs each occurred at a frequency of 0.5% in the study population. Lastly, the very rare synonymous variant detected at position c.387C>T (p.Ser129=) (rs144828000) has to date not been described in the context of the *NAT2* acetylation status, and occurred in 1% of the study cohort. Further assessment of the protein and enzymatic activity of these SNVs was not within the scope of this study. Future studies, however, may investigate these less commonly detected SNVs, whether ‘in silico’ or otherwise.

There are limited data available on the most commonly occurring *NAT2* haplotypes in the mixed ancestry population of the Western Cape, although numerous studies have been performed in other population groups. Fuselli et al. demonstrated a preponderance of NAT2*4 (37%), NAT2*7B (24%) and NAT2*5B (23%) in 13 American and two Siberian populations.^32^ Our study showed a similar predominance of NAT2*4 (28%) and NAT2*5B (27.4%), while the frequency of NAT2*7B was much lower (3.8%). While the wild type (NAT2*4) was also the most commonly occurring haplotype in East Asians,^33^ Loktionov et al. described NAT2*5B to be the most common haplotype in black South Africans,^34^ followed by NAT2*6A and NAT2*12A (both 16.8%, vs 21.5% and 7.5% in our study population). In contrast to our findings, the wild type NAT2*4 was shown to occur less frequently in black South Africans (13.4%).^34^ In a cohort of 62 black and 54 mixed ancestry South Africans, where 17% were found to be fast acetylators^35^, haplotype frequencies did not differ significantly between the two population groups (unpublished). The frequency of *NAT2* haplotypes obtained in this study serve as a priori information to strengthen future haplotype predictions by computational algorithms such as PHASE, specifically in this mixed ancestry population.

The good correlation between PHASE and molecular haplotyping methods demonstrated in our mixed ancestry population is similar to previous studies in other populations. A study by Golka et al. in a German population, where the statistical program PHASE version 2.1.1 was used to predict haplotypes from genotypes by analysing seven *NAT2* SNVs commonly observed in the Caucasian population, concluded that this program unambiguously derived haplotypes in over 99% of cases.^36^ Similarly, other studies have shown that these computational algorithms (including PHASE) compare well with molecular haplotyping, and could thus be used as an alternative method for genetic phasing. Sabbagh and Darlu (2005) investigated the performance of several computational approaches in estimating individual haplotype phases in five different population groups (Spanish, United Kindom Caucasians, black South Africans, Koreans and Nicaraguans).^17^ They compared the results obtained from the computational programs with those obtained through direct molecular haplotyping and found that even the incorrectly predicted haplotypes did not result in phenotypic changes, resulting in a complete correlation of the acetylation phenotypes in all five population groups. The population group in which computational haplotype inference displayed the highest error rate was black South Africans, although error rates were always low (less than 4%). The postulated reasons include the high number of ambiguous genotypes as well as the low rate of linkage disequilibrium between the various SNVs in this sample, compared with the other four population groups.^17^ The authors suggest studying these data before computational haplotyping is employed, to attempt to predict the accuracy and reliability with which haplotypes will subsequently be inferred in such cases.

The single incorrectly predicted phenotype (intermediate to slow) may be clinically significant. However, the computational program flagged the predicted haplotypes as being uncertain; in the clinical scenario, such a flag could serve as a prompt to confirm the haplotype by molecular methods instead of relying on PHASE.

In our current setting, *NAT2* acetylation status is chiefly investigated by special request once the patient has hepatic complications of isoniazid therapy. Apart from the adverse drug reactions that could be avoided by individualising isoniazid dosage according to *NAT2* phenotypes, incorrect dosage may contribute to the growing prevalence of multi-drug resistant tuberculosis.^37^ The unavailability of routine isoniazid genotyping could thus be considered a major limitation of current tuberculosis treatment protocols. N-acetyltransferase 2 genotyping should ideally be performed on all patients before commencing isoniazid therapy. The cost of genetic investigations as well as the limited infrastructure in the South African healthcare setting make it difficult to apply haplotyping without the PHASE computational approach. This approach could be supported by specific investigation of incompletely explored SNVs and the development of a high throughput multiplex assay targeting common SNVs relevant to the population being investigated. Further studies are also recommended to investigate and monitor the isoniazid safety and treatment outcome in patients undergoing treatment for tuberculosis with different *NAT2* acetylation phenotypes.

### Limitations

Due to budgetary and time constraints, only a small sample size of 100 was used. The ARMS PCR could not be set up for the following variants which may have improved the correlation: c.590G>A, c.857G>A, c.341T>C and c.803A>G.

### Conclusion

PHASE accurately predicted the *NAT2* acetylation phenotype in 92 of 93 samples (99%) from genotypic data in our mixed ancestry study population, and could be used as an alternative to molecular methods to individualise isoniazid therapy in our high tuberculosis burden setting. Data obtained from this study may also be potentially used to predict haplotypes in future patients from this population group.
